# Agent-based models under uncertainty

**DOI:** 10.12688/f1000research.135249.2

**Published:** 2023-08-31

**Authors:** Vladimir Stepanov, Scott Ferson

**Affiliations:** 1 Institute for Risk and Uncertainty, University of Liverpool, Liverpool, England, L69 7ZX, UK

**Keywords:** epistemic uncertianty, agent-based modelling, intervals

## Abstract

**Background:** Monte Carlo (MC) is often used when trying to assess the consequences of uncertainty in agent-based models (ABMs). However, this approach is not appropriate when the uncertainty is epistemic rather than aleatory, that is, when it represents a lack of knowledge rather than variation. The free-for-all battleship simulation modelled here is inspired by the children’s battleship game, where each battleship is an agent.

**Methods:** The models contrast an MC implementation against an interval implementation for epistemic uncertainty. In this case, our epistemic uncertainty is in the form of an uncertain radar. In the interval method, the approach occludes the status of the agents (ships) and precludes an analyst from making decisions about them in real-time.

**Results:** In a highly uncertain environment, after many time steps, there can be many ships remaining whose status is unknown. In contrast, any MC simulation invariably tends to conclude with a small number of the remaining ships after many time steps. Thus, the interval approach misses the quantitative conclusion. However, some quantitative results are generated by the interval implementation, e.g. the identities of the surviving ships, which are revealed to be nearly mutual with the MC implementation, though with fewer identities in total compared to MC.

**Conclusions:** We have demonstrated that it is possible to implement intervals in an ABM, but the results are broad, which may be useful for generating the overall bounds of the system but do not provide insight on the expected outcomes and trends.

## Introduction

An agent-based model (ABM) is a model for simulating the actions and interactions of autonomous agents (either individual or collective entities such as organisations or groups) with the intention of observing the emergent behaviour of the whole system. ^1^ ABMs are temporally explicit, usually with a fixed unit of time referred to as ticks. An ABM consists of its agents, with each agent being an autonomous discrete unit with its own aims, priorities and actions. ^1^ Agents can also vary between themselves. The agents can be cooperative or prioritise their individual goals. An example of cooperative agents could be a coalition to lift a heavy object, while individualistic agents could be animals competing for scarce resources.

ABMs generally do not incorporate any epistemic uncertainty, such as imprecision or doubt about the agent’s properties or behaviours. ^2^ It is argued that this kind of uncertainty can be modelled with distributions ^3^ and Monte Carlo (MC) methods. With the MC approach, an ABM is executed a vast number of times with different parameters, which reflect the possible values that the parameter could take. ^4^^–^^7^ This approach is computationally expensive, though there are strategies to reduce the number of computations, *e.g.*, Latin hypercube sampling. ^8^

Besides attempts at reducing the computational cost of MC, some attempts try to reduce the computational cost of the model directly, *e.g.* in discrete event simulation, the introduction of “time buckets” which are intervals of time in which multiple events can occur. ^9^ The equivalent strategy for an ABM would be a coarsening strategy that increases the fixed time step. This might be considered as introducing temporal epistemic uncertainty into an ABM, but as it handles this uncertainty in a simplistic way (in other words, by ignoring it) and with the simulation losing some of its detail depending on the magnitude of the time-step increase, which may not be desirable. ^10^ As an example of losing detail, consider logging your position in daily life. If you log every 15 minutes, you might have 5 entries: home (0), travel (15), shop (30), travel (45) and home (60). However, if you log every hour, you will log: home (0) and home (60). Hence, you have lost detail, which in some applications may be critical.

However, introducing non-time-based epistemic uncertainty into an ABM is not as simple as described above. For this implementation we will use intervals, which are arguably the simplest representation of epistemic uncertainty. ^11^ The intervals will be used to model a free-for-all battleship simulation with uncertain radar ranges (inspired by the children’s game), showing that it is possible to use intervals to model battleship behaviour and status, but this approach occludes the status of the agents and precludes decision making about them.

One perspective against the use of intervals in ABM is that the parameters the intervals represent can be easily approximated with uniform distributions. However, this approach with uniform distributions acts as though the parameter is varying randomly, which may not be true. ^12^^,^^13^ Though reasonable, there are some instances where combining two approximated intervals in this manner can cause problems, *e.g.*, when A = [0.2, 0.4]; B = [0.3, 0.5], the product AB is [0.06, 0.2], but it will also indicate that there is a higher probability of it being a central value in the result if A and B are based on uniform distribution. Similarly, the sum of A and B will be [0.5, 0.9], but if approximated *via* uniform distributions it will also contain a central peak in this range. There is no justification for this higher central probability if the same calculation is performed without approximation but just utilising intervals. ^12^^,^^13^ Thus, approximating with uniform distributions is not always appropriate.

**Figure 1.**
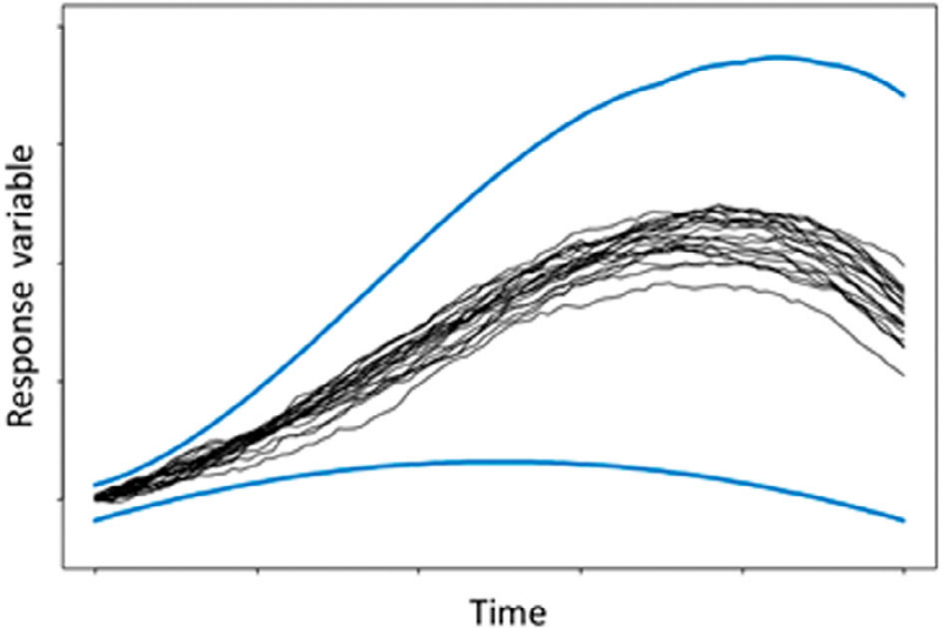
Graphical representation of Monte Carlo realisations (shown in black) against possible outer bounds (blue). Reproduced with permission from original.

Therefore, it can be argued that a direct implementation of intervals in ABMs will avoid the above-mentioned problem. Additionally, a direct implementation should have a lower computational cost than MC and potentially avoid the problem that MC does not explore the full range of possibilities. Furthermore, it is argued that MC is poor at identifying extreme events, as can be observed in Figure 1, where it shows that most of the MC runs are concentrated in the central region of the outer bounds. ^2^ However, the figure may exaggerate the convergence of the runs in respect to the outer bounds (indicated by the blue lines), but it helps to illustrate the centralisation effect of many random variables.

## Model configuration

The model is constructed in Python (v3) ^14^ (RRID:SCR_008394), with the following packages: numpy ^15^ (RRID:SCR_008633), enum, ^14^ numbers, ^14^ csv, ^14^ matplotlib ^16^ (RRID:SCR_008624), time, ^14^ tqdm. ^14^ The discrete simulation (global seed 0) can last up to 250 time-steps, during which the agents move about and interact with one another. The agents representing battleships are set in an X,Y grid (102 × 102) where the edges of the grid wrap-around so that a ship leaving the top of the grid reemerges at the bottom of the grid, and vice versa. Likewise, the right side wraps to the left side and vice versa, which is a toroidal or Pac-Man topology. ^17^ The grid is randomly populated with 60 ships that will try to sink all other ships that they detect.

Each ship is located in a grid point depicted as a dot in the grid, with the ship’s radar as a circle around the dot. The ships move at constant velocities during the simulation. Each ship is given a random velocity between 1 and 6 grid units per time step. The initial direction of travel is random (determined by the movement generator with seed 2021), but each ship is turning in circular arcs across the grid, changing direction by π /30 radians at each time step. Each ship also has a maximum number of missiles, (uniformly) randomly generated from 10 to 60 missiles, that it launches at enemy ships whenever detected by radar whose range is (uniformly) randomly initialised for each ship between 2 to 27.5 grid units. Additionally, the ships are limited by the number of ships it can target in each time step (randomly generated from 2 to 16) ordered by proximity, with preference given to the ships closest to the ship firing the missiles. In an ideal world the declared radar range would always detect enemy ships within its boundaries. However, in a more realistic world a ship’s radar varies across its range. Close to the ship, the radar detects enemies perfectly (certain radar), but for enemies farther away, detection is uncertain. These two ranges are represented as two concentric circles: the inner certain radar range, and the larger uncertain radar range).

At each time step, each ship moves to another grid point determined by its velocity, and from this new location detects other ships within its certain radar range and *may* detect those within its uncertain range. Once all targets are identified, the ship fires missiles at them as long as the upper bound of the interval for the missiles remaining is greater than zero. Due to uncertainty about the radar range, a ship may or may not detect a target ship within its firing range. In such a case, the firing of missiles is uncertain. If the ship detects the target it would fire, but if it does not detect the target it would not fire. This means that, when representing the state at the next moment of time, we consider both possibilities: that the ship fires a missile and that the ship does not fire the missile. In the former case, the ship’s complement of missiles decreases and the target may experience damage. In the latter, neither occurs. This is necessary as the ships know their complement of missiles but as the simulation is from the viewpoint of the analyst, the analyst does not.

Additionally, the ship can have less than the maximum number of targets and can even have zero targets if no other ships are located in its radar range. However, the number of missiles per target remains constant at three missiles, but if three missiles are not available, then the ship will fire any remaining missiles at the target. The ships’ actions are concluded by assessing any possible damage (hits) to itself that has been suffered due to the other ships having fired their missiles. Positive hits are determined from the missiles *via* a binomial distribution, with the chance of failure set as 0.5. If such hits sink the ship, its existence is negated. If the hits would surely not have sunk the ship, it continues to exist. If the hits *could but might not* sink it, the ship’s existence is represented as uncertain. The two states are depicted by the line type (solid line - ship exists; dashed line - ship with uncertain existence). Three *certain* missile hits will sink any ship.

The model code can be found on GitHub. ^18^

## Possible model scenarios

The model keeps track of each ship’s state of existence and its remaining missiles. A ship is sunk when the number of missile hits exceeds the maximum possible hit threshold (3). There can be uncertainty about a ship’s existence, with this state a result of a missile from another uncertain ship increasing this ship’s hit count over the maximum possible hit threshold. The hit count is represented as an interval with the lower bound representing the total number of certain hits and the maximum bound representing the possible number of hits. Similarly, a ship’s missile count is represented as an interval once the ship has fired a missile at a ship located in its uncertain radar range. Here the upper bound represents the total number of certain missiles that the ship potentially has, while the lower bound represents the number of missiles it has left if it fired at the ship in the uncertain radar range.

**Figure 2.**
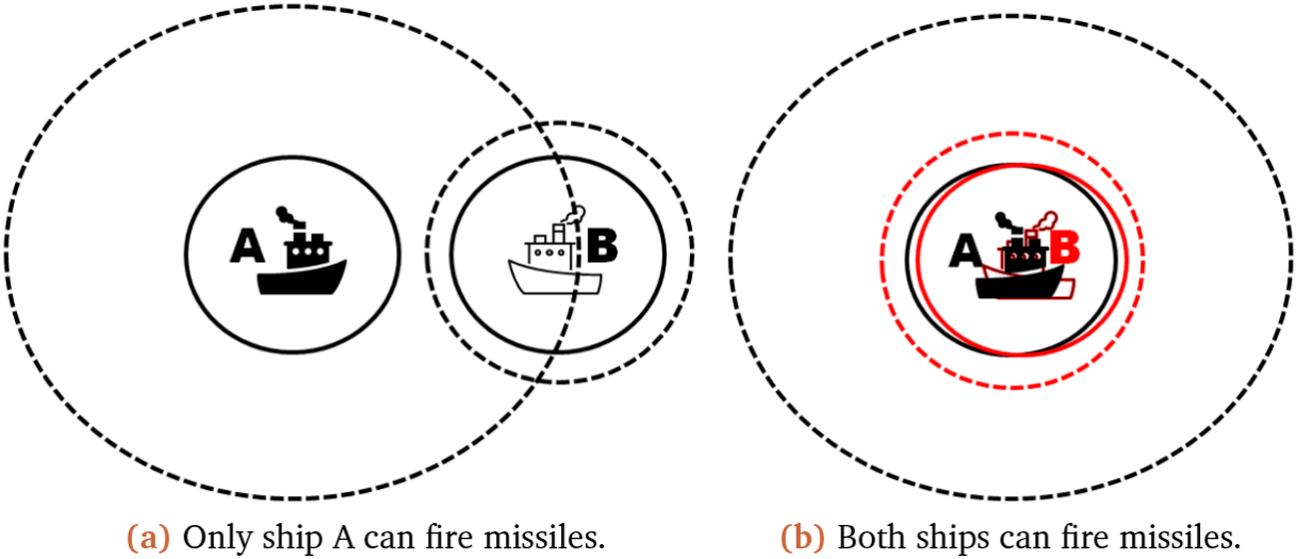
Scenarios illustrating the possible ship outcomes. The full line represents the certain radar range of the ship, while the dashed line represents the outer edge of its uncertain radar range.

To justify our decision on the interval implementation we have provided some scenarios to explain our reasoning. In our first scenario only one ship can fire at the other, while the second scenario shows the effect of uncertain existences. In these scenarios, the solid line within the Figures 2 represents the outer edges of the certain radar (if we see a ship here, we definitely see a ship), while the dashed line represents the outer range of the uncertain part of the radar (we are unsure that we have seen a ship there). We will represent missiles left as ***M***, missiles fired as ***m***, previous missiles hit as ***H***, and missiles hit in this time segment as ***h***. We will use subscripts to represent the ship that these values belong to.

It is shown in Figure 2a that only one ship (A) can fire missiles at the target ship (B). Thus ship A options can be listed as: it sees ship B, it does not see ship B; leading to ship A being able to fire and not fire its missiles, which would lead to an updated missile count for fire as ***M − m*** and not fire as ***M***. We can summarise the missiles left for ship A with an interval [ ***M − m, M***]. It is important to note that if ship A does not exist, the outcome is equivalent to not firing the missiles as the argument is if the ship does not exist, then it cannot detect ship B, thus it will not be able to fire missiles. The other ship, Ship B, is the one that is getting fired upon by ship A, tracks the number of missiles ***h*** that have hit it. If the number of missiles that have hit the ship is zero, then the total number of hits remain unchanged at ***H*** (also true if ship A does not exist and so (ship A) does not have the capacity to fire missiles), otherwise the number of hits is ***H + h***. Thus the total number of hits can be represented as [ ***H, H + h***]. As mentioned before, a ship’s existence is tied to the number of certain hits it has taken, which means that ship B will not sink as long as the existence of ship A is uncertain or the missiles are fired into the uncertain radar range of the attacking ship.

In the second scenario ( Figure 2b) we explore the case where both ships are located in their certain range but the ships themselves are uncertain about their existence. Here the actions of ship A are mirrored for ship B, therefore only a detailed explanation for ship A will be provided. Ship A will fire its missiles if ship B exists, and it will not fire if ship B does not exist. Hence the remaining missiles for ship A will be $\left[M_{A}-m_{A},M_{A}\right]$ and the remaining missiles for ship B will be $\left[M_{B}-m_{B},M_{B}\right]$ . Ship A’s hit count will both increase by the number of missiles that hit ship A and stay the same as we are unsure if B has fired missiles as if B exists then surely it has fired the missiles, while if B does not exist then no missiles can be fired, thus $\left[H_{A},H_{A}+h_{A}\right]$ . As only the upper bound (of the hit count) is increased due to uncertainty, ship A will continue existing, no matter how high the upper bound gets. The same is true for ship B’s hit count $\left[H_{B},H_{B}+h_{B}\right]$ .

In summary, the scenarios demonstrate that an uncertain ship cannot sink a certainly existing ship, as the missiles fired from such a ship are uncertain. Thus only the upper bound of the hit count for the other ship is increased. When the hit count’s upper bound exceeds the ship’s upper existence limit, the previously certainly existing ship becomes uncertain about its existence, but it does not (certainly) sink. Recall that uncertainty about a ship’s existence means that we don’t know whether it has been sunk or not. This can be expanded to include that two uncertain ships cannot sink each other and that only a certainly existing ship can sink another certainly existing ship. Though the scenarios represent possible edge cases that can occur in the simulation, they can also illustrate the in-between states satisfactorily. These scenarios have been presented to aid in the understanding of the model; as the ships do not communicate with an external observer, thus we do not know the state of the ships or the number of missiles remaining.

## Model results

The emphasis of this investigation was to explore the effect of an imprecise radar range and how it could be propagated inside an ABM. ^19^ To be able to propagate the effect of the imprecise radar range other uncertainties have to be added for the model to function, *e.g.* missile count and ship existence. We also varied the radar range for all the ships to explore the differences, while keeping the same seed for the random generator in all the experiments, in other words, we changed the parameter that affected the radar range while preserving the model architecture, with the results shown in Table 1.

**Table 1.** Table summarising the model outcomes based on the relative radar range. Square brackets denote interval ranges. All results are shown for seed 0. Relative radar range 1 represents the original radar range.

Relative radar range	Precise		Imprecise	
	Ships	Missiles	Ships	Missiles
0.5	3	53	[0,15]	[62, 660]
0.75	4	79	[0,15]	[0, 574]
1	2	9	[0, 6]	[22, 234]
1.5	2	36	[0, 5]	[80, 230]
2	1	24	[0, 4]	[12, 254]
4	1	15	1	[3, 12]

In the first simulations, with no epistemic uncertainty, i.e. when the ships see other ships in their radar perfectly, denoted Precise in Table 1, each ship’s original radar range was selected uniformly randomly to be between 2 and 27.5. This original setting is denoted by relative radar range of 1. Subsequent simulations collectively reduced or increased these ranges. For instance, for relative radar range of 2, the upper limit was doubled to 53. This meant that ships that originally had a relatively smaller radar range maintained their rank when their radar range was doubled. As we are interested in verifying the methodology and not the behaviour of the ships, we can utilise the same seed as multiple simulations are not needed, as the seed ensures that the same actions should be performed in each simulation. Additionally, even though the radar range was changed, the other parameters (starting number of missiles, initial velocity) were preserved. At the end of the simulation, with the original radar range, there are two ships and nine missiles remaining. Halving the range yields three ships with 53 missiles, while increasing the range by a factor of two gives one ship left with 24 missiles ( Table 1). It can be seen that for the radar range of 0.75, the number of surviving ships is higher than for radar range 1, which is expected, but it is also higher than the number of surviving ships when the radar range is 0.5. The cause could be fewer ships sunk in the initial moments of the simulation as each ship sees fewer targets, leading to more initially surviving ships, thus leading to more targets in the simulation. This requires more random values, thus possibly shifting the outcome; in other words the greater number of surviving ships could be a consequence of the increased usage of the random number generator. This results in the need for multiple simulations and reveals the necessity of controlling the random numbers, which will be considered below.

With an imprecise radar, the results are different as we get no definitive numbers but rather intervals (denoted by square brackets) for the possible solution ( Table 1). With the original radar range we simulate that there are in the interval [0, 6] ships at the end and that they have [22, 234] missiles between them. Halving the radar range we increase the possible number of ships left to 15 [0, 15] with a larger possible number of missiles left [82, 660]. On the other hand increasing the radar range decreases the number of ships left [0, 4], but the overall possible number of missiles left [12, 254] is increased, though the interval starts with less fully known missiles.

Contrasting the two results, we observe that imprecise radar results are more ambiguous than their precise counterpart (for this particular seed) but they do follow the same general trend, *i.e.* as radar decreases there are more ships and missiles available. It is also important to note that as the imprecise radar decreases, the uncertainty increases in the number of ships and missiles remaining. This increase in uncertainty could be due to the increase in uncertain ships as they cannot be sunk and thus more uncertain ships mean there are more uncertain missiles fired.

Table 1 shows that, although the current implementation of intervals is rather crude, it can also be argued that there is no feasible alternative for propagating epistemic uncertainty in an ABM. Thus, propagating with intervals is similar to propagating the worst-case and the best-case scenarios simultaneously. Given the available information, it is not possible to decrease the span of the results without additional knowledge. For example, consider a ship that has uncertainty about a target. The current model accounting for this epistemic uncertainty has the ship both firing and not-firing its missiles. Alternatively, the ship’s policy could be to fire a *reduced* number of missiles if it is unsure about a target, hence reducing the missiles’ interval. In such a case, if the modeller knows about the ship’s policy, the missile count interval and its epistemic uncertainty could then be reduced.

The results of Table 1 show that the imprecise (interval) implementation follows the same pattern as the precise results, but these results do not show that the interval implementation is a viable alternative, as the current precise radar results do not account for other possible radar ranges. Hence, for a more fair comparison, the precise radar ranges need to be varied to compensate for the uncertain radar range, which can be achieved *via* MC simulation. For simplicity we used the minimum (perfect radar range from the interval implementation) and maximum values (maximum possible radar range from the interval implementation) as the two possible ranges for the outer bounds of each uniform distribution from which each ship’s radar range is generated, as the MC implementation assumes ships see other ships perfectly in its entire radar range. The simulations were implemented in a straightforward MC approach, where the radar range varieties are produced in Python ^14^ with the numpy.random.default_rng class with random seed 220 and the uniform function of this class. The model itself remains unchanged, with the same random generators and parameters.

To ensure a fair comparison between MC and the interval implementation, we must be able to generate the same sequence of events. Because the comparison is set between the results of the model, where each action in the model is determined by a random generator ( *e.g.*, the initial velocity of the ships, and the outcome that a fired shot found its target), a common strategy is to set the generator to repeat the list of random numbers in each run by setting the same start point for the generator at the simulation start. However, as this model is agent-based, this is not sufficient to guarantee that the actions taken by agents are the same when the ship’s radar ranges are varying. In other words, a ship may perform more actions as a result of a bigger radar range, thus requiring additional random numbers for the additional actions. As all the random numbers created from a set generator can be expressed as a list, this means more uses of the random generator leads to a longer list. Additionally, if each agent utilises the same generator, this means an additional action in the simulation by one agent can shift the random numbers used in other agents, thus possibly generating an alternative outcome as the agents may behave differently.

To prevent inconsistencies from divergences arising from such additional actions and to prevent unexpected path changes when varying the radar range, a predetermined path for each agent was generated. This was achieved by generating each path as if the simulation were being performed with each ship surviving for the full duration of the simulation time and recording these paths outside of Python. In our case, this is not a concern because ships all turn at a constant π /30 radians, as explained above. This framework using predetermined paths can be used in simulations where ship paths can include randomness. This framework ensures that the agents do not deviate due to shifting random values. However, divergence between our “All random” results and “Preset path, all missiles binomial hit” was observed, even though we are utilising a constant turn path. This was found to be due to a truncation effect from the recorded path values having fewer decimal points.

The final aspect that can change alongside the radar range changing is the outcome from the launched missiles: a ship with a larger radar range can fire earlier or detect a ship that it couldn’t detect beforehand. Thus, as there are more opportunities for the ship to fire missiles, the outcome of the barrage can shift, *e.g.* when before some particular missile may sink a particular ship now it may not, thus resulting in our previously generated random values to be used earlier and a demand for more random values to be generated. Thus, another measure that we have implemented to reduce the potential uncertainty lies in how a missile hit is determined. Our original implementation models missile hits binomially with 0.5 chance of failure. To reduce this uncertainty, we changed the original binomial to no longer have a failure state, thus ensuring that any missile fired always hits the target, *i.e.* the missiles fired can not miss. This modification is referred to as “all missiles fired hit”.

With these random generator control measures, the simulation results should only differ due to the ship radar range changes, thus allowing us to compare the two methodologies’ outcomes as equivalently as possible. Additionally, we have run the model without some of the control factors to demonstrate the variability without these control measures. The MC runs are collated as intervals into the Table 2 for easier comparison between the interval implementation (as this implementation outputs a pre-made interval) and the MC one.

**Table 2.** Table showing the resulting differences between interval and Monte Carlo (MC) implementation and which control measure generates the greatest possible number of surviving ships. From the results, it can be seen that the interval implementation encompasses the MC one, with nearly all the Ship IDs found in the interval implementation occurring in the MC one.

	Total ships (interval)	Ship IDs (interval)	Total ships (MC)	Ship IDs (MC)	Shared IDs
All random	[0, 12]	1, 16, 21, 25, 30, 31, 35, 36, 37, 50, 52, 57	[0, 5]	0-3, 5-6, 8-19, 21-26, 28-31, 34-44, 46-50, 52-57, 59	1, 16, 21, 25, 30, 31, 35, 36, 37, 50, 52, 57
Random path, all missiles fired hit	[0, 6]	0, 13, 16, 36, 41, 48	[0, 5]	0, 11, 14, 16, 24, 30, 31, 35, 36, 37, 41, 48, 50, 56, 59	0, 16, 36, 41, 48
Preset path, all missiles binomial hit	[0, 25]	0, 4, 6, 11-14, 21, 24, 25, 28, 30, 32, 37, 41, 42, 44, 47, 48, 52, 54-57, 59	[4, 18]	0-50, 52-59	0, 4, 6, 11-14, 21, 24, 25, 28, 30, 32, 37, 41, 42, 44, 47, 48, 52, 54-57, 59
Preset path, all missiles fired hit	[0, 15]	0, 13, 24, 30, 32, 35-38, 41, 44, 48, 54, 57, 59	[1, 10]	0, 2, 6, 13, 24, 25, 30, 35-38, 41, 42, 44, 48, 50, 54, 55, 57, 59	0, 13, 24, 30, 35-38, 41, 44, 48, 54, 57, 59

The results in Table 2 show that the interval implementation encompasses the MC results for the total number of ships left. It is also important to note that the general pattern for the remaining number of ships is shared between the two implementations, *i.e.* more ships survive on a pre-set path with binomially distributed hits for the missiles; while the smallest number of ships survive with a random path and all missiles fired hit.

As a further review, the ship IDs are recorded to be compared between the two methods to ensure that the two methods simulate a similar outcome. Table 2 shows that the interval method has less variety in the possible ship’s ID compared to MC, as the interval method can only simulate one outcome under the set seeds. Thus, we need to check that the ship IDs generated from the interval method are found in the MC method to demonstrate that the ship ID outcomes are possible. Going back to Table 2, we can observe that nearly all the ship IDs found in the interval method can be found in the MC method, apart from Ship 13 (random path, all missiles fired hit) and Ship 32 (pre-set path, all missiles fired hit), but they may still be found present in the MC simulation if more MC runs were undertaken.

**Figure 3.**
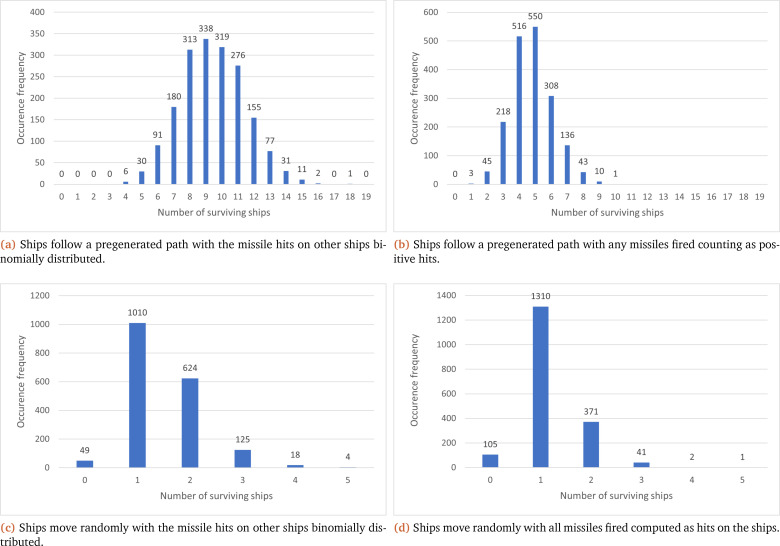
Collection of graphs showcasing the number of surviving ships at the end of each simulation (X axis) and their overall frequency (Y axis) under the MC method.

In this vein, as multiple iterations of the model are necessary for MC, there is additional data generated compared to the interval implementation (see endpoint results: Table 2), which can be collated to show the total number of occurrences for the number of ships that survived at the end of the simulation. This can be seen in Figure 3, where each sub-figure corresponds to one of the four previously discussed scenarios. Sub-figures (a) and (b) show that a predetermined path has a higher frequency for the number of surviving ships (with peak occurrence values at nine and five respectively) compared to their associated random path scenario (where both peak values are at one ship). Figure 3 also shows that the paths that the ships follow in the simulation have the greatest effect on the shape of the results; with a preset path ((a) and (b)) displaying results similar to a normal distribution, while a random path ((c) and (d)) coincides more with our expectations (one ship surviving and more ships surviving being less common). Another common factor shown in Figure 3 is that the number of surviving ships at the end of the simulation is higher when they are binomially distributed ((a) and (c)) compared to every missile fired hitting the target ((b) and (d)).

In summary, the results show that interval implementation encompasses the MC results for the high-level results (total number of ships left surviving), but there are fewer ship IDs in total compared to MC. Thus, the interval implementation may be good for finding the possible extreme values, while MC for the expected outcomes. However, it is important to note that extreme events may be more important as failure events are usually located in this region.

## Conclusion

We have demonstrated that it is possible to implement and propagate intervals directly in an ABM, with the understanding that the interval endpoints represent the possible extreme values. Further, we show that MC is an ideal method for finding the expected outcomes and trends, as well as being simple to implement across various ABM models. However, in this case, it is poor at handling epistemic uncertainty due to assuming an interval can be represented as a uniform distribution but that is necessary for the MC method to work. Thus, other methods are needed for epistemic uncertainty, however they are also not without their drawbacks.

One of the drawbacks is that the answers may be vacuous (in the battleships example the answer is presented as an interval), while MC depends on how the results are collated at the end ( *e.g.* enumerate the number of occurrences for 0 ships left; the number of times one ship is left … ). Additionally, new rules may be added to the battleship interval implementation for some aspects to generate smaller intervals ( *e.g.* how missiles are fired if they are uncertain about a target), but as the other aspects cannot be adapted it stands that the underlying problems with this type of implementation will still remain.

Therefore, a direct implementation of intervals and propagating the uncertainty about a value with their use is not recommended as the results generated are rather broad and do not provide additional help in decision making, though it may be useful in generating the overall bounds of the system. Furthermore, depending on the model applications an interval model may be preferable as its computational time is lower compared to MC once the model is built.

## Data Availability

Zenodo: Battleship Monte Carlo Results for comparison against Interval Implementation. https://doi.org/10.5281/zenodo.7990753. ^19^ This project contains the following underlying data:
• PathsX.csv (Input file for X coordinates of ships using a predetermined path) • PathsY.csv (Input file for Y coordinates of ships using a predetermined path) • ResultsSPathBinMC.txt (contains the preset path and Binomially distributed hits) • ResultsRPathBinMC.txt (contains the random path and Binomially distributed hits) • ResultsSPathATMC.txt (contains the preset path and all missiles fired hits) • ResultsRPathAllTMC.txt (contains the random path and all missiles fired hits) • Scenario.txt (The minimum radar range for interval implementation) • ScenarioM.txt (The maximum radar range for interval implementation) Data are available under the terms of the Creative Commons Attribution 4.0 International license (CC-BY 4.0).

## Software availability

Source code: https://github.com/vvstepanov/Battleship/tree/v1.0a

Archived source code at the time of publication: https://doi.org/10.5281/zenodo.8113453 ^18^

License: MIT.
