# Supplementary material for: Agent-based models under uncertainty
Source: F1000Res. 2024 Mar 14;12:834. Originally published 2023 Jul 14. [Version 3] doi: 10.12688/f1000research.135249.3 (PMC10988201; doi:10.12688/f1000research.135249.3)
Supplement: Peer review history

# Reviewer report: Reviewer response for version 3

**DOI:** 10.5256/f1000research.163616.r255979

**Published:** 2024-04-02

**Authors:** Barbara Llacay

**Affiliations:** 1 University of Barcelona, Barcelona, Spain

I consider that the authors have appropriately addressed my comments.

Is the rationale for developing the new method (or application) clearly explained?

Yes

Is the description of the method technically sound?

Yes

Are the conclusions about the method and its performance adequately supported by the findings presented in the article?

Yes

If any results are presented, are all the source data underlying the results available to ensure full reproducibility?

Yes

Are sufficient details provided to allow replication of the method development and its use by others?

Yes

Reviewer Expertise:

Agent-based simulation, artificial markets

I confirm that I have read this submission and believe that I have an appropriate level of expertise to confirm that it is of an acceptable scientific standard.

# Reviewer report: Reviewer response for version 2

**DOI:** 10.5256/f1000research.154859.r211384

**Published:** 2023-11-08

**Authors:** Barbara Llacay

**Affiliations:** 1 University of Barcelona, Barcelona, Spain

The paper discusses the use of intervals as a method to address epistemic uncertainty in agent-based models. Uncertainties related to agent behaviours or properties can be represented using distributions and Monte Carlo (MC) methods, but MC can be computationally expensive. The authors propose using intervals as a straightforward way to represent epistemic uncertainty, demonstrating their use in a battleship simulation with uncertain radar ranges.

 Some comments:

- There is a typo in the keywords ('uncertianty')
- Related work: The use of intervals to deal with epistemic uncertainty can be related to the use of fuzzy logic. Given that other authors have presented fuzzy agent-based models in fields where uncertainty is prevalent (some examples are listed below), could the authors clarify in what way their work is related to this type of models?
  - Raoufi, M. and Fayek, A.R. (2021), "Hybrid fuzzy Monte Carlo agent-based modeling of workforce motivation and performance in construction", Construction Innovation, Vol. 21 No. 3, pp. 398-416. https://doi.org/10.1108/CI-03-2020-0045
  - Hassan, S., Garmendia, L., Pavón, J. (2007). Agent-Based Social Modeling and Simulation with Fuzzy Sets. In: Corchado, E., Corchado, J.M., Abraham, A. (eds) Innovations in Hybrid Intelligent Systems. Advances in Soft Computing, vol 44. Springer, Berlin, Heidelberg. https://doi.org/10.1007/978-3-540-74972-1_7
  - Yildiz, B. and Çağdaş, G. (2020). Fuzzy logic in agent-based modeling of user movement in urban space: Definition and application to a case study of a square,
    Building and Environment, Vol. 169. https://doi.org/10.1016/j.buildenv.2019.106597
- The certain and the uncertain radar ranges are defined as concentric circles. How are their respective radii determined?
- When describing the model configuration, the authors say that "Positive hits are determined from the missiles via a binomial distribution, with the chance of failure set as 0.5. If such hits sink the ship, its existence is negated. If the hits would surely not have sunk the ship, it continues to exist. If the hits could but might not sink it, the ship’s existence is represented as uncertain." --> How do the authors assess whether "the hits would surely not have sunk the ship" or whether "the hits could but might not sink it"? Please clarify.
- The authors say that "that only a certainly existing ship can sink another certainly existing ship", However, is it not possible that a certainly existing ship can sink an uncertain ship?

Is the rationale for developing the new method (or application) clearly explained?

Yes

Is the description of the method technically sound?

Yes

Are the conclusions about the method and its performance adequately supported by the findings presented in the article?

Yes

If any results are presented, are all the source data underlying the results available to ensure full reproducibility?

Yes

Are sufficient details provided to allow replication of the method development and its use by others?

Yes

Reviewer Expertise:

Agent-based simulation, artificial markets

I confirm that I have read this submission and believe that I have an appropriate level of expertise to confirm that it is of an acceptable scientific standard, however I have significant reservations, as outlined above.

## References

[1] : Hybrid fuzzy Monte Carlo agent-based modeling of workforce motivation and performance in construction. *Construction Innovation* .2021;21(3) : 10.1108/CI-03-2020-0045 398-416 10.1108/CI-03-2020-0045

[2] : Enhancing temozolomide antiglioma response by inhibiting O6-methylguanine-DNA methyltransferase with selected phytochemicals: in silico and in vitro approach. *3 Biotech* .2023;13(12) : 10.1007/s13205-023-03821-7 385 10.1007/s13205-023-03821-7 37928438 PMC10622385

[3] : Fuzzy logic in agent-based modeling of user movement in urban space: Definition and application to a case study of a square. *Building and Environment* .2020;169: 10.1016/j.buildenv.2019.106597 10.1016/j.buildenv.2019.106597

# Reviewer report: Reviewer response for version 1

**DOI:** 10.5256/f1000research.148359.r193841

**Published:** 2023-08-22

**Authors:** Josie McCulloch

**Affiliations:** 1 University of Leeds, Leeds, England, UK

The authors present an interesting method of using interval computations to understand epistemic uncertainty in an agent based model, with the goal of counteracting the problems with Monte-Carlo methods not being so suited to this kind of uncertainty.

 The paper is well written, but there are a few points that could be better explained.

 In the abstract, I'm not sure what the authors mean by "the interval approach misses the quantitative conclusion".

 On page 4, why can a ship's radar reach 27.5 grid units? The text states that a ship occupies a grid cell, so it's not clear there's any advantage to being able to see half a cell.

 Also on page 4, it states that a ship will fire a missile if the lower bound of the interval for the remaining missiles is above zero. Why does a ship have uncertainty in how many missiles it has? Is it the model that has uncertainty in how many missiles the ship has, and therefore what the ship then does?

 In addition, if there is another ship in uncertain radar range, then I don't think the current ship can be said to have both fired and not fired its missiles. I think it would be more accurate to say it may or may not have fired its missiles.

 Also, a typo on page 4, it should be "missiles fired as m".

 On page 5, the authors say the first simulations have no epistemic uncertainty. It would be useful to summarise how this is so. Do they have no uncertainty in their radar?

Is the rationale for developing the new method (or application) clearly explained?

Yes

Is the description of the method technically sound?

Yes

Are the conclusions about the method and its performance adequately supported by the findings presented in the article?

Yes

If any results are presented, are all the source data underlying the results available to ensure full reproducibility?

Yes

Are sufficient details provided to allow replication of the method development and its use by others?

Yes

Reviewer Expertise:

Agent-based models, interval computations

I confirm that I have read this submission and believe that I have an appropriate level of expertise to confirm that it is of an acceptable scientific standard, however I have significant reservations, as outlined above.

# Response

**Published:** 2023-08-27

**Authors:** Vladimir Stepanov

**Affiliations:** University of Liverpool, UK

Thanks to Prof McCulloch for her thoughtful and careful comments.  They have been valuable to us.  We have numbered the points she has raised for easy reference.

*1)In the abstract, I'm not sure what the authors mean by "the interval approach misses the quantitative conclusion".*

 We have changed “the” to “that” as we feel that helps to clarify that an interval implementation output is an interval. This interval may not be small, which may be unrealistic.

*2)On page 4, why can a ship's radar reach 27.5 grid units? The text states that a ship occupies a grid cell, so it's not clear there's any advantage to being able to see half a cell.*

 Thank you for pointing this out. As a result, we have changed the terminology from grid cell to grip point to better reflect the code implementation and to reduce confusion.

*3)Also on page 4, it states that a ship will fire a missile if the lower bound of the interval for the remaining missiles is above zero. Why does a ship have uncertainty in how many missiles it has? Is it the model that has uncertainty in how many missiles the ship has, and therefore what the ship then does?*

*In addition, if there is another ship in uncertain radar range, then I don't think the current ship can be said to have both fired and not fired its missiles. I think it would be more accurate to say it may or may not have fired its missiles.*

 In addition to Vladik’s reply we have also added a clarifying statement that the ship doesn’t have uncertainty about how many missiles it’s fired, but the simulation does.  That is, the analyst who is running the simulation.

*4)Also, a typo on page 4, it should be "missiles fired as m".*

 Corrected.

*5)On page 5, the authors say the first simulations have no epistemic uncertainty. It would be useful to summarise how this is so. Do they have no uncertainty in their radar?*

 We have added the following clarifying statement: “i.e. when the ships see other ships in their radar perfectly,” – to remove confusion.

# Reviewer report: Reviewer response for version 1

**DOI:** 10.5256/f1000research.148359.r187524

**Published:** 2023-07-31

**Authors:** Vladik Kreinovich

**Affiliations:** 1 Department of Computer Science, The University of Texas at El Paso, El Paso, Texas, USA

The problem that this paper solves is very ubiquitous. It is related to the fact that in many real-life situations, we need to make decisions under uncertainty. Sometimes, we know the probabilities of different alternatives; in this case, we can use usual Monte-Carlo simulation techniques. However, in many practical situations, we only have partial information about the corresponding probabilities. Frequently, all we know is the interval of possible values of some quantities, and we have no information about the probability of different values within this interval. In such situation of interval uncertainty, some practitioners assume that there is a uniform distribution on this interval, If the objective is to select a single distribution out of all possible distributions on the given interval, this assumption makes perfect sense: since we have no reason to believe that some values are more probable than others, it makes sense to assume that all the values are equally probable, i.e., that we have the uniform distribution. However, as is well known -- and as the authors convincingly show -- this assumption can lead to a drastic under-estimation of the resulting uncertainty. Instead, the authors propose to explicitly carry on intervals of possible values at each moment of the simulation -- and when moving to the next moment of time, consider all possible transitions which are consistent to the available interval information.

 The authors illustrate this idea on the example of a battleship simulation inspired by the well-known children's game. The simulation is interesting and the results are interesting, but the text needs a few corrections and some clarification editing.

 Minor typo: on p. 3, 0.02 should be 0.20.

 p. 4, first full paragraph, the ship fires only if the lower bound on the number of remaining missiles is larger than 0. This needs explanation, I did not expect it. We may not know during simulation whether the ship has any missiles left or not -- since in the previous moment of time, it was not clear whether the ship noticed the adversary and fired the missile. In this case, for us, the lower bound for the number of missiles is 0. However, in the actual run, the ship knows how many missiles it has left, so if a missile is left, it can fire it -- while this phrase seems to not allow this possibility.

 The next phrase is even more confusing: the ship has both fired the missiles and not fired the missile. I know that everyone likes this confusing description of Schroedinger's cat that journalists use when they want to entertain the reader, but here the authors are trying to explain, so why not make it clearer. I think what the authors want to say is that in this case, when forming the state at the next moment of time, we take into account both possibilities: that the ship fires a missile and that the ship does not fire the missile.

 Very minor thing: the authors use it's when it should be its: e.g., its radar range (it's means it is, it is an exception to the general rule of adding apostrophe and s to a noun).

 p. 5, last paragraph before the section titled "Model results", line 1: it is not clear why an uncertain ship cannot sink a certainly existing ship: uncertain ship means, e.g., that without our general description, in some possible histories, the ship is already destroyed. However, the fact that the ship is uncertain at this moment of the simulation means that in some possible histories, the ship is still there -- in which case it can sink another ship.

 p. 5, line 3 from the bottom: I am not sure why the fact that the same seed was utilized, we do not need multiple simulations: using the same seed means that we select one random value out of many, if we selected a different seed and a different value, we could get a different result. In this case, the conclusions based on one simulation may not be applicable to other cases -- and to make general conclusions, we do need additional simulations. For the same reason, the last phrase in the first (incomplete) paragraph on p. 6 is not clear.

Is the rationale for developing the new method (or application) clearly explained?

Yes

Is the description of the method technically sound?

Yes

Are the conclusions about the method and its performance adequately supported by the findings presented in the article?

Yes

If any results are presented, are all the source data underlying the results available to ensure full reproducibility?

Yes

Are sufficient details provided to allow replication of the method development and its use by others?

Yes

Reviewer Expertise:

Interval computations

I confirm that I have read this submission and believe that I have an appropriate level of expertise to confirm that it is of an acceptable scientific standard, however I have significant reservations, as outlined above.

# Response

**Published:** 2023-08-18

**Authors:** Vladimir Stepanov

**Affiliations:** University of Liverpool, UK

Thanks to Prof Kreinovich for his thoughtful and careful comments.  They have been valuable to us.  We have numbered the points he has raised for easy reference.

*1) Minor typo: on p. 3, 0.02 should be 0.20.*

 Thank you. This has been corrected.

*2)p. 4, first full paragraph, the ship fires only if the lower bound on the number of remaining missiles is larger than 0. This needs explanation, I did not expect it. We may not know during simulation whether the ship has any missiles left or not -- since in the previous moment of time, it was not clear whether the ship noticed the adversary and fired the missile. In this case, for us, the lower bound for the number of missiles is 0. However, in the actual run, the ship knows how many missiles it has left, so if a missile is left, it can fire it -- while this phrase seems to not allow this possibility.*

 We have changed the word “lower” to “upper” in the confusing sentence.  This allows for the possibility of firing missiles, as expected.

*3)The next phrase is even more confusing: the ship has both fired the missiles and not fired the missile. I know that everyone likes this confusing description of Schroedinger's cat that journalists use when they want to entertain the reader, but here the authors are trying to explain, so why not make it clearer. I think what the authors want to say is that in this case, when forming the state at the next moment of time, we take into account both possibilities: that the ship fires a missile and that the ship does not fire the missile.*

 We have changed the original sentence from:

 If the ship fires at a ship in the uncertain radar range, the ship fire is uncertain, i.e. the ship has both fired the missiles and not fired the missiles. 

 To this:

 Due to uncertainty about the radar range, a ship may or may not detect a target ship within its firing range. In such a case, the firing of missiles is uncertain.  If the ship detects the target it would fire, but if it does not detect the target it would not fire. This means that, when representing the state at the next moment of time, we consider both possibilities: that the ship fires a missile and that the ship does not fire the missile. In the former case, the ship’s complement of missiles decreases and the target may experience damage.  In the latter, neither occurs.

*4)Very minor thing: the authors use it's when it should be its: e.g., its radar range (it's means it is, it is an exception to the general rule of adding apostrophe and s to a noun).*

 Corrected.

*5)p. 5, last paragraph before the section titled "Model results", line 1: it is not clear why an uncertain ship cannot sink a certainly existing ship: uncertain ship means, e.g., that without our general description, in some possible histories, the ship is already destroyed. However, the fact that the ship is uncertain at this moment of the simulation means that in some possible histories, the ship is still there -- in which case it can sink another ship.*

 We have clarified the paragraph that explains how a ship becomes uncertain about its existence by changing the explanation from:

 In summary, the scenarios demonstrate that an uncertain ship cannot sink a certainly existing ship, as when the upper bound for the hit counts exceeds the ship’s limit, the previously certainly existing ship becomes uncertain about its existence.

 To this:

 In summary, the scenarios demonstrate that an uncertain ship cannot sink a certainly existing ship, as the missiles fired from such a ship are uncertain. Thus only the upper bound of the hit count for the other ship is increased. When the hit count’s upper bound exceeds the ship’s upper existence limit, the previously certainly existing ship becomes uncertain about its existence, but it does not (certainly) sink.  Recall that uncertainty about a ship’s existence means that we don’t know whether it has been sunk or not.

*6)p. 5, line 3 from the bottom: I am not sure why the fact that the same seed was utilized, we do not need multiple simulations: using the same seed means that we select one random value out of many, if we selected a different seed and a different value, we could get a different result. In this case, the conclusions based on one simulation may not be applicable to other cases -- and to make general conclusions, we do need additional simulations. For the same reason, the last phrase in the first (incomplete) paragraph on p. 6 is not clear.*

 We agree. We have modified the paragraph discussing the issue in two places: the middle and at the end. The additional part in the middle, explains why we have used one seed (we were testing the methodology and not the actual behaviour of those particular ships). Our edit at the end of the paragraph, clarifies that the simulation can still have variations even when using the same seed, leading to further testing explained at a later stage in the text. We hope that the changes improve clarity.
